# Physical activity measured using wearable activity tracking devices associated with gout flares

**DOI:** 10.1186/s13075-020-02272-2

**Published:** 2020-08-03

**Authors:** Nada Elmagboul, Brian W. Coburn, Jeffrey Foster, Amy Mudano, Joshua Melnick, Debra Bergman, Shuo Yang, Lang Chen, Cooper Filby, Ted R. Mikuls, Jeffrey R. Curtis, Kenneth Saag

**Affiliations:** 1 University of Alabama at Birmingham, 1720 2nd Avenue South, Birmingham, AL 35294 USA; 2 University of Nebraska Medical Center and VA Nebraska-Western Iowa Health Care System, Omaha, NE USA

**Keywords:** Interactive voice response system, Smartphone application, Gout flares

## Abstract

**Objective:**

To determine the feasibility and validity of using wearable activity trackers to test associations between gout flares with physical activity and sleep.

**Methods:**

Participants with physician-diagnosed gout, hyperuricemia (≥ 6.8 mg/dl), current smartphone use, and ≥ 2 self-reported flares in the previous 6 months were enrolled. Physical activity, heart rate, and sleep data were obtained from wearable activity trackers (Fitbit Charge HR2). Daily compliance was defined by the availability of sufficiently complete activity data at least 80% of the day. Associations of weekly gout flares with sleep and activity were measured by comparing flare-related values to average sleep and steps per day. We used mixed linear models to account for repeated observations.

**Results:**

Forty-four participants enrolled; 33 met the criteria for minimal wear time and flare reporting, with activity tracker data available for 60.5% of all total study days. Mean ± SD age was 48.8 ± 14.9 years; 85% were men; 15% were black; 88% were on allopurinol or febuxostat, and 30% reported ≥ 6 flares in the prior 6 months. Activity trackers captured 204 (38%) person-weeks with flares and 340 (62%) person-weeks without flares. Mean ± SD daily step count was significantly lower (*p* < 0.0001) during weeks with gout flares (5900 ± 4071) than during non-flare periods (6972 ± 5214); sleep however did not differ.

**Conclusion:**

The pattern of wear in this study illustrates reasonable feasibility of using such devices in future arthritis research. The use of these devices to passively measure changes in physical activity patterns may provide an estimate of gout flare occurrence and duration.

**Trial registration:**

NCT, NCT02855437. Registered 4 August 2016

## Background

Gout flares are one of the main outcomes of urate lowering therapy in randomized control trials [1, 2]. Flare symptoms are variable and can last from hours to days depending on their severity and the gout management strategy. Over time, gout flares lead to a significant decline in function and disability [3, 4]. Gout also is associated with disturbances in sleep quality and quantity [5]. One of the challenges in gout studies is the inability to capture flare occurrence in a timely and accurate fashion since flares primarily occur external to the health care setting. With the advent and widespread uptake of digital technologies and a validated patient-reported gout flare definition [6], there is an opportunity to innovate around improved methods for gout flare capture [7]. However, systems that require regular proactive patient input (e.g., weekly or daily patient gout flare diaries) are not optimal given the participant burden required and the resulting likelihood of patient reporting attrition over time. Less obtrusive means of remote flare capture that build on common digital technologies are of high interest. Passive data capture using such technologies might facilitate feasible and valid capture of gout flares.

In recent years, wearable activity tracker devices have been widely used and adopted for personal activity monitoring. Their popularity is associated with ease of use, ability to wirelessly synchronize with smartphones, and the ability to record multiple types of data in real time. Wearable activity trackers have also been incorporated more frequently into clinical research given their capability to capture daily activities including step counts and sleep duration and depth. Wearable activity trackers have also been used in studies assessing physical activity in individuals with musculoskeletal diseases such as osteoarthritis, rheumatoid arthritis, and spondyloarthritis [8, 9]. As one example, Fitbit® devices (Fitbit Charge HR2) have widespread popularity, and as of 2016, the Fitbit device platform holds a significant share of the wearable tracker market [10]. This proprietary device has been utilized in many clinical trials [11] and evaluated in many validity studies [12].

Despite the increased use of the wearable activity trackers in various research studies, the suitability of data retrieved from such devices for research purposes remains somewhat unsettled [13]. One challenge with such devices is defining methods to assess a patient’s wear compliance or “wear time.” While removing the device creates a missing data problem, only a few studies reported specific criteria for wear time [14] or data completeness. Other studies only used the patient self-reported wear times [15] or through wear time journals [16].

In this study, we evaluated the feasibility of using a wearable activity tracker, Fitbit®, to capture the impact of gout flares on physical activity and sleep as part of a 6-month, prospective pilot study designed to examine preferences and feasibility to capture patient-reported gout flares. We also examined the associations of physical activity and sleep changes with flares. Finally, given uncertainties on the best methods to handle missing data from the wearable activity tracker, since participants might remove it for periods of time, and given the lack of a widely accepted definition of consistent wear of the tracker devices, we evaluated different definitions for the completeness of data capture and their impact on our results.

## Participants and methods

### Study population and design

After approval by the relevant Institutional Review Boards, the study was conducted in rheumatology clinics at two academic medical centers, the University of Alabama at Birmingham (UAB) and the University of Nebraska Medical Center (UNMC, Omaha, NE), from September 2016 to March 2018. Eligibility criteria included adults, age ≥ 18 years, smartphone ownership, rheumatologist-diagnosed gout, self-report of ≥ 2 gout flares in the previous 6 months, and hyperuricemia (serum urate level ≥ 6.8 mg/dl) measured within 3 months of screening. We evaluated sleep and physical activity using a wearable activity tracker, Fitbit® Charge HR2 (Fitbit Inc., San Francisco, CA, USA), provided as part of the study to each participant. We captured gout flares using scheduled queries sent through a custom software application (“StudyBuddy”) developed at UAB, or via an interactive voice response system (IVR) [7]. Each participant was provided the wearable activity tracker at the initial visit with the StudyBuddy and Fitbit apps installed and tested on their mobile phones. We requested participants to wear the device consistently for 6 months except when charging it or bathing. For the StudyBuddy application to capture all physical activity and sleep data, the wearable activity tracker was synced automatically via Bluetooth (in the background), or manually every 4 days. Beyond scheduled 12-week clinic visits or phone calls, we did not provide reminders aimed at improving adherence in order to be more pragmatic in our study design.

### Physical activity, sleep, and heart rate measurement using wearable activity trackers

Raw data on sleep duration, physical activity (step count), and heart rate were obtained from the wearable activity tracker in minute-level increments. All Fitbit data was obtained automatically by the StudyBuddy application through direct queries to the developer’s application-programming interface (API) that occurred at least once weekly.

### Tracker feasibility and wear measurements

Heart rate data alone is sometimes used to classify whether the tracker device is being worn or not. To ascertain the impact on wear time classification by adding in information about step count and sleep, we evaluated a variety of definitions of wear time compliance. First, any minute where heart rate, step count, or sleep data was available classified that minute as wearing the device. We then evaluated the impact of imputing wear time in 15-, 30-, and 60-min intervals. For example, for the 60-min imputation, if any minute in an hour was deemed as having compliant wear time, the entire 60 min of that hour would be considered to be compliant wear time. Hours were then aggregated into 24-h intervals, partitioning days at 4 pm (rather than at midnight) to avoid subdividing the usual period for sleep.

We then classified different compliance patterns using a 24-h period as the unit of analysis. A “Compliant wear with sleep” designation was assigned when the device was worn at least 80% of the 1440 possible minutes in a day. For participants choosing not to wear the device at night, a 24-h period was classified as having “Compliant wear without sleep” when the device was worn at least 80% of 960 possible minutes (16 h, excluding 8 h during which the participant was assumed to be sleeping). The 24-h periods where no wearable activity tracker data was available were classified as “No health tracker data”, and all other patterns were described as “Partial wear” days.

### Gout flare measurements

Participants reported gout flares by completing survey questions via weekly scheduled StudyBuddy application questions or programmed IVR calls at a time selected by each participant. These survey questions were derived from the validated gout flare instrument [6]. Two definitions were used for gout flares: (1) a single-item definition, where the participant reported whether or not he was having a flare (yes/no), and (2) a more stringent validated definition by Gaffo et al. [6] based on fulfilling at least 3 of 4 possible criteria (patient-defined gout flare, pain at rest score of > 3 on a 0–10 point numerical rating scale, self-reported presence ≥ 1 swollen joint, and presence ≥ 1 warm joint). The self-report flare instrument captured weekly reports of gout flares as well as each flare start date. Since we measured wearable activity tracker data and aggregated from its native minute-level precision to 24-h periods, the gout flare reported data was converted from a weekly recall period to 24-h periods and aligned temporally with the wearable activity tracker data.

#### Data management and statistical analysis

The pattern of wear time for each participant over the course of the 6-month study was illustrated using a heat map and colored as (1) Compliant wear with sleep days, (2) Compliant wear without sleep days, (3) Partial wear days, and (4) No health tracker data days. We measured the association of flare with mean step count and sleep duration collected from the wearable tracker device, comparing flare days to non-flare days using both the single-item gout flare and the more stringent validated gout flare definition. We then re-analyzed our results across different wear compliance groups to assess whether the completeness of tracker data meaningfully impacted the associations observed with gout flare.

Baseline characteristics of study participants were summarized with counts for categorical variables and means and standard deviation (SD) for continuous variables. For comparisons of activity tracker wear compliance data, descriptive statistics with proportions were used. To study changes in sleep and step counts on days when the participant reported a gout flare, we used a mixed linear model to account for repeated observations and to adjust for potential confounders. An alpha of 0.05 was used to determine significance, and all analyses were performed with SAS (version 9.4, SAS, Cary, NC).

## Results

Forty-four participants were enrolled with 33 meeting the minimum criteria for wear time of the activity tracker and flare reporting. One participant withdrew immediately post-randomization, and five were lost to follow-up. Out of a total of 6572 days with activity tracker data, the effective sample size including compliant and partial compliant wear patterns included 3978 days of observation among 33 subjects. When we merged the Fitbit data with StudyBuddy/IVR data, we generated 3426 days for 33 subjects out of the total 44 participants between the two data sets (75%). Participants were mostly well-educated, middle-aged men with average gout disease duration of 10 years. Over one-half reported ≥ 4 gout flares in the 6 months prior to study enrollment (Table 1).

**Table 1:** Baseline characteristics of study participants (*n* = 33)

	***N*** (%) or mean (SD)
Age (years)	48.8 (14.9)
Sex	
Men	28 (84.9)
Race	
White*	27 (81.8)
Black	5 (15.2)
Other*	1 (3.0)
Education level	
Less than high school	1 (3.0)
High school or G.E.D.	8 (24.2)
Some college (junior college, technical degree, etc.)	13 (39.4)
4-year college degree or higher	11 (33.3)
Age of first gout flare (years)^†^	38.1 (18.6)
Duration of gout (years)^†^	10.5 (8.2)
Number of flares prior 6 months	
1–3	16 (48.5)
4–6	7 (21.2)
> 6	10 (30.3)
Gout medication use	
Urate lowering therapy	29 (87.9)
NSAID or colchicine	21 (63.6)
Prednisone	12 (36.4)
Number of smartphone apps on cellphone^†^	20.6 (17.2)

*NSAID* non-steroidal anti-inflammatory; urate lowering therapy included allopurinol, febuxostat, or probenecid; not mutually exclusive to other gout medications

*Two declared Hispanic ethnicity; ^†^missing data = 1

### Wearable activity tracker device feasibility and wear time imputation

**Fig. 1:**
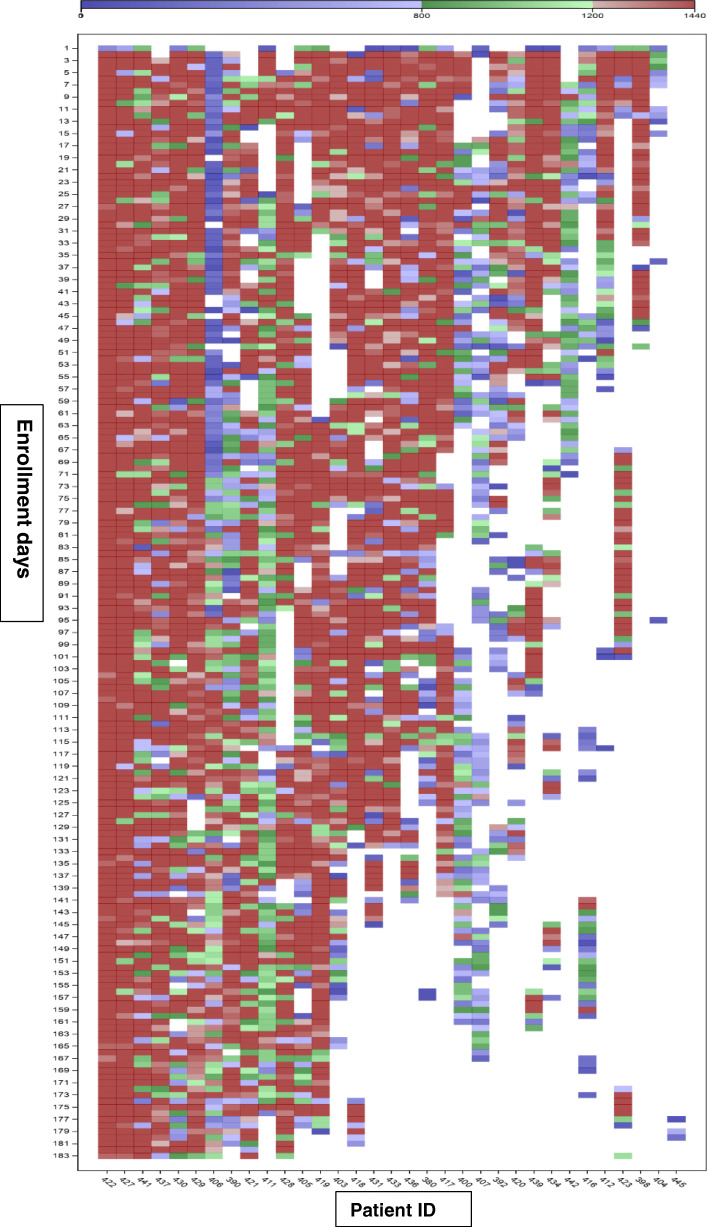
Heat map showing daily compliance with wearing the health tracker device. Compliance analysis for each participant is shown with data in each column reflecting a participant; each row is a person-day in the study. Red = compliant wear (≥ 80%) with sleep data; green = compliant wear (> 80%) without sleep data; blue = partial wear; white = not wearing

Analyzing days when the activity tracker was worn (60.5% of all study days), 68% of days were classified as Compliant wear with sleep (red), 7% as Compliant wear without sleep (green), and 25% as Partial wear (blue) (Fig. 1). If we consider only the first 3 months of data, censoring participation at 84 days, 66% of all study days had activity tracker data, with 68% of days considered as Compliant with sleep data, 6% Compliant without sleep, and 26% of days as Partial wear.

The impact of adding step count and sleep data to the heart rate data, as well as imputing wear time in 15-, 30-, and 60-min increments, is shown in Table 2. If only heart rate data was used with no imputation, a total of 4,182,919 min would have been classified as the participant wearing the device. Once step count and sleep data was incorporated, the number of wear minutes increased by 2.6% to 4,292,447 study minutes.

**Table 2:** Effect of aggregating heart rate, step count, and sleep data, and imputation of wear time in 15-, 30-, and 60-min increments, to classify time wearing the activity tracker device

Imputation*	Heart rate minutes	% increase from imputation of wear time	Step count minutes	% increase from imputation of wear time	Sleep minutes	% increase from imputation of wear time	Composite of any (heart rate, step count, sleep) minutes	% increase wear time above heart rate minutes and after imputation of wear time**
**Raw data, no imputation**	4,182,919	Referent	828,780	Referent	1,155,986	Referent	4,292,447	2.6%; referent
15-min imputation* intervals	4,354,035	+ 4.1%	2,342,250	+ 182.96%	1,204,320	+ 4.2%	4,472,685	2.7%; 4.2%
**30-min imputation intervals**	4,426,740	+ 5.8%	2,877,690	+ 247.2%	1,255,920	+ 8.6%	4,552,950	2.9%; 6.1%
**60-min imputation intervals**	4,522,500	+ 8.1%	3,424,920	+ 313.3%	1,357,500	+ 17.4%	4,657,020***	3.0%; 8.5%

Heart rate minutes = minutes with heart rate data; step count minutes = minutes with steps data; sleep minutes = minutes with sleep data

*Imputation to the specific interval was performed if there was any value (heart rate, steps, or sleep) in 1 min was non-missing

**The first number refers to the increase in wear time related to the use of the 3 data types vs. only step count data, shown in the first column of the same row; the second number refers to the gain in wear time related to imputation compared to the first row

Imputing wear time to the nearest 15, 30, or 60 min increased wear minutes assigned using only heart rate data by approximately 4, 6, and 8%, respectively, compared to no imputation. A similar effect was seen for the composite of heart rate, step count, and sleep minutes. Overall, using the composite of heart rate, step count, and sleep data, and imputing to the nearest 60 min, 8.5% more study minutes (4,657,020) were classified as wearing the activity tracker. When stratifying these effects according to whether the person-day was classified as Compliant wear vs. Partial wear, the effect of imputation was more profound on Partial wear days (mean increase of 23.0% of wear minutes) but still meaningful for Compliant wear days (mean increase of 6.0% of wear minutes) (Supplemental Table).

### Associations with gout flares

When we analyzed the mean change in step count on days with Compliant wear data, there was a difference in step count on days with single-item gout flare compared to non-flare days (Table 3). Importantly, our definitions for Compliant wear mattered in terms of the association with gout flare on that day. A mean difference of − 841 steps was observed on study days with Compliant wear with sleep data (*p* < 0.0001), − 675 steps on days including Compliant wear with sleep + Compliant wear without sleep (*p* = 0.0007), and − 472 steps on days with all available data (i.e., days with any non-missing data, including Partial wear data and ignoring wear patterns) (*p* = 0.02). In a secondary analysis based on validated flares, the step count change results were similar (Table 3). There was an average difference of − 864 steps on days with Compliant wear with sleep (*p* = 0.0001) and − 718 steps on days with Compliant wear with sleep + Compliant wear without sleep (*p* = 0.0008). Likewise, there was a change by − 396 steps noted on flares compared to non-flare days when we examined all available data, although this was not significantly different (*p* = 0.05).

**Table 3:** Average daily step count differences by level of trackable wear compliance and gout flare classification

Wearable tracker compliance classification	Gout flare classification definition	Participants, flare days	Participants, non-flare days	Mean ± SD step count on flare days	Mean ± SD step count on non-flare days	Adjusted difference * (***p*** value)
**Compliant wear with sleep**	Single item	Subjects = 25 Total days = 442	Subjects = 29 Total days = 1791	5900 ± 4071	6973 ± 5214	Δ = − 841 (*p* < 0.0001)
**Compliant wear with sleep + Compliant wear without sleep**	Single item	Subjects = 29 Total days = 504	Subjects = 30 Total days = 1991	6171 ± 4096	7007 ± 5173	Δ = − 675 (*p* = 0.0007)
**All available Fitbit data, ignoring wear pattern**	Single item	Subjects = 31 Total days = 666	Subjects = 30 Total days = 2547	5330 ± 4090	6236 ± 5032	Δ = − 472 (*p* = 0.0121)
**Compliant wear with sleep**	Validated	Subjects = 21 Total days = 383	Subjects = 29 Total days = 1791	5930 ± 3983	6973 ± 5214	Δ = − 864 (*p* < 0.0001)
**Compliant wear with sleep + Compliant without sleep**	Validated	Subjects = 24 Total days = 439	Subjects = 30 Total days = 1991	6173 ± 3984	7007 ± 5173	Δ = − 718 (*p* = 0.0008)
**All available Fitbit data, ignoring wear pattern**	Validated	Subjects = 27 Total days = 559	Subjects = 30 Total days = 2547	5482 ± 3977	6236 ± 5032	Δ = − 396 (*p* = 0.0542)

Compliant wear with sleep = > 80% of 1440 min recorded. Compliant wear without sleep = > 80% of 960 min = recorded; all available data = partial wear (= wear minutes > 60 < 800 recorded) + compliant wear with sleep + compliant wear without sleep

*To estimate *p* value, mixed linear models were used to adjust estimation for repeated observations for an individual

In the sensitivity analysis where flare days were restricted to those meeting the validated gout flare definition, a total of 559 days met the validated gout criteria definition vs. 666 days in the single-item definition, accounting for approximately 16% gain in flare days using the latter approach, more sensitive definition. A numeric difference in step count associated with gout flare was observed when comparing the single-item gout flare vs. validated flare definition. On days with compliant wear with sleep, flare based on the validated definition was associated with 184.82 fewer steps compared to the single-item gout flare definition, although this difference did not achieve statistical significance (*p* = 0.71). There were no significant changes in sleep duration during single-item (8.09 ± 3.35) or validated flare vs. no flare periods (8.2 ± 3.22) (*p* = 0.7) (data not shown).

## Discussion

Using a commercially available health activity tracker device that captured data on step count and sleep, we detected a significant difference of 841 steps in the daily step count on days where a gout flare occurred versus days where no gout flare was reported. However, we did not observe a change in sleep duration during flares. Using the more specific validated gout flare definition, there was a numerically larger effect on step count. Eighty-two percent of the participants met criteria for minimal wear time of the activity trackers, with data available for 60.5% of total study days. Considering days for which we had any health activity tracker data, 75% of those days reached our definition of compliant wear time, indicating reasonable feasibility of wearable tracker use in gout clinical studies. We also increased our analyzable data when we used a composite of heart rate, sleep, or step count data to identify wear time. Further data gains were seen with imputation of wear time to 60-min intervals compared to shorter intervals, suggesting that the methods used for defining tracker wear in such studies may have a modest effect on study results. Furthermore, a more liberal and sensitive definition of gout flare, a single-item definition based solely on patient report, increased the flare days captured by the trackable devices.

The feasibility of using wearable activity trackers has been reported in various studies of other rheumatologic and chronic diseases with heterogeneous results. A recent meta-analysis assessed the adherence and effectiveness of wrist wearable trackers to increase physical activity in rheumatic diseases. That study reported a mean wear time of more than 90% in 3 studies; however, all had shorter mean duration of only 10 weeks. A 16-week study looking at physical activity adherence through self-monitoring intervention with a personalized goal among postmenopausal females found a median of ≥ 10 h/day adherence to wear in 95% of the intervention days [17]. In another study of children with juvenile idiopathic arthritis [18], a wrist activity tracker used to capture physical activity data was worn on 72% of the days in the intervention period. We found a somewhat lower compliance pattern than in these other studies. These differences in wear compliance might be secondary to the longer 6-month duration of our study when compared to other studies as well as the differences in study objectives and diseases being monitored. The fact that our investigation represented a more real world, pragmatic experience lacking close monitoring for compliance, is another difference compared to other studies where regular follow-up with weekly calls from the research team and monthly face-to-face meetings [19] or scheduled study visits [20] likely contributed to the higher adherence observed in those reports.

Despite the increase of use as well as the many validity studies with wearable activity trackers [14, 21, 22], definitions for compliance with wearing the device, and standards for sufficient completeness of the activity tracker data to permit valid analysis, are not well described. In past studies, cutoff points ranging from 10 to 30 min of zero wear counts per day were used to identify interrupted wear [23, 24]. Variable cutoff points from 1 to 10 h per day to consider that the information was complete enough to permit analysis have been used previously, with no particular recommendations [23, 25, 26]. A methodologic study comparing 4 wear time algorithms suggested that the most stringent criteria used adversely impacted the sample size [27]. In particular, the most stringent algorithm defined minimal wear time as 12 h per day and allowed no more than at least 20 consecutive minutes of non-wear, while the most liberal algorithm allowed up to 60 consecutive minutes of non-wear. Significant differences were observed in wear time among different algorithms ranging between 960 and 779 min/day. These findings parallel observations from our study where the adoption of overly stringent cutoffs (imputing wear time to ≤ 30 min) would have been problematic resulting in substantial data loss, whereas imputation of wear time to 60-min intervals yielded a considerable increase in overall wear time data. We also demonstrated that, as we expected, heart rate data was most useful to evaluate wear time, but there were gaps of minutes where data was missing (e.g., due to band being worn too loosely on the wrist), such that the composite of minute-level step count, heart rate, and sleep data appears to provide a more comprehensive measure of wear time than using heart rate alone. Gout flares are a subjective and sometimes poorly captured outcome measure in gout research; hence, they are critical yet problematic as an endpoint in clinical trials. Thus, there is substantial interest in identifying methods to better define flares that are not dependent on direct patient report. Since gout flares are associated with patient-reported reductions in physical activity and disturbed sleep [4, 5], better ways to capture these potential surrogates for gout flares are of high interest, particularly using passive methods that are not dependent on individuals filling out questionnaires. In our study, we demonstrated objective evidence of reduced step counts with gout flares, as has been previously reported in other rheumatic diseases using wearable health trackers [28]. When comparing changes in physical activity in all wear patterns between the single-item, self-reported flare and the more specific validated gout flare definition, there was a greater magnitude in the reduction in step count during flare days when the more rigorous gout flare definition was used. This finding could reflect the greater specificity of the validated flare definition, with the expectation that those flares were more severe, and less severe flares (as were sometimes captured using the broader definition) yielded a smaller impact on step counts. Larger studies are needed to confirm our findings using a range of gout flare definitions. Our finding that sleep duration was not affected by gout flare might be secondary to one of the known weaknesses of wearable activity trackers in overestimating sleep duration, as has been reported [29], or simply that a gout flare does not meaningfully affect sleep. It is also possible that sleep duration is not the optimal metric to assess in relation to gout flares, but rather, quality and stages of sleep (e.g., deep sleep, REM) could be more meaningfully affected by such flares. This topic should be examined in future studies to analyze stage of sleep and associated duration of time in each stage, rather than merely sleep minutes.

This pilot study also highlighted potential challenges*.* Although many participants were highly compliant with the tracker device, there were sometimes lengthy gaps of non-wear, limiting the sensitivity of the device and suggesting the importance of more real-time monitoring of participant wear patterns during the course of future studies aimed at outcome assessment.

Also we were not able to fully address other causes behind reduced physical activity such as pain for any other reason, the participant’s occupation, and activity variations during different days of the week. A few study participants encountered technical issues with device-smartphone synchronization as well as issues with charging their devices; these concerns should be offset in future studies as available technology becomes even more commonplace and easier to use. Based on these observations, we recommend that future studies leveraging this technology coordinate near real-time data access to effectively monitor participation and intervene as gaps in data become apparent. In a larger 250+ person RA study [30], based on experience from this study, we incorporated some changes in the study design by introducing more active daily data monitoring as well as accounting for occupational status, hobbies, and days of the week that could act as confounding factors.

Since our study was a proof-of-concept study, our main objective was to examine the association of gout flares (single-item and validated) with physical activity. Future larger studies including those with subset analysis, such as examining response by types of urate lowering therapy, will be important.

Additionally, history of health states affecting sleep such as insomnia was not obtained, which might have resulted in substantial variability in sleep patterns that could not be effectively controlled for in the study, making it more difficult to detect the impact of a gout flare. Self-reported flare data was collected weekly, making the identification of the gout flare duration potentially challenging given the reliance on patient recall. An increased frequency for the collection of flare data might be considered in future studies to offset these concerns, at minimum collecting data daily after a flare has been captured. Lastly, we did not protocolize patient engagement to maximize wear time, but this design feature was deliberate to allow us to better ascertain feasibility of a largely “hands-off” data collection approach.

## Conclusion

Our study provides valuable and novel insight about the potential of using wearable activity trackers to passively measure changes in physical activity patterns that might provide estimates of disease activity and duration of gout flares. We found that heart rate data alone as measured by health activity tracking devices was not an ideal measure to determine whether participants are wearing such a device. Our method of imputation of wear time to 60-min intervals captured in 1-min increments for all three data types (heart rate, step count, and sleep) yielded more robust results than using heart rate data alone. The possibility of wearable technology being used as an adjunct to patient reports versus an independent tool is yet to be further validated but remains a goal. If validated further, this could free patients from having to frequently answer questions and provide patient-reported outcome (PRO) data, or at least, reduce data collection burden often encountered by studies. Future work will be needed to assess the application of the passive data from wearable activity trackers to monitor gout flares while examining new interventions in gout, but these results are a promising first step to support the feasibility of incorporating these elements into such a study to better capture flares, especially using passive data sources alone, or infrequently supplemented by active data collection through PRO instruments. The ability of the activity tracker device to not merely classify but to predict a flare remains an opportunity for the future.

## Supplementary information

**Additional file 1: Table S1.** Completeness of Fitbit® Data across Data Types, and after Imputing Wear Time Across Various Intervals. Effect of imputation using 24 h person-days as unit of aggregation.

## Data Availability

The datasets generated and/or analyzed during the current study are not publicly available due to complexity of data coding and data transformations needed for analyses but are available from the corresponding author on reasonable request.

## Abbreviations

IVR: Interactive voice messaging response system
StudyBuddy: Smartphone mobile application
API: Application-programming interface
RCTs: Randomized controlled trials
REM: Rapid eye movement
ULT: Urate lowering therapies
NSAID: Non-steroidal anti-inflammatory
PRO: Patient-reported outcome
