# Supplementary material for: Physical activity measured using wearable activity tracking devices associated with gout flares
Source: Arthritis Res Ther. 2020 Aug 3;22:181. doi: 10.1186/s13075-020-02272-2 (PMC7398057; doi:10.1186/s13075-020-02272-2)
Supplement: Supplementary file 1 — Additional file 1: Table S1. Completeness of Fitbit® Data across Data Types, and after Imputing Wear Time Across Various Intervals. Effect of imputation using 24 h person-days as unit of aggregation. [file 13075_2020_2272_MOESM1_ESM.docx]

**Supplementary table:**

**Table S1. Completeness of Fitbit® Data across Data Types, and after Imputing Wear Time Across Various Intervals**

**Effect of imputation using 24 hour person-days as unit of aggregation**

|  | **Heart Rate Minutes** | **% Increase from Imputation  of wear Time** | **Step Count Minutes** | **% Increase from Imputation of wear Time** | **Sleep Minutes** | **% Increase from Imputation of wear Time** | **Composite of any (Heart Rate, Step Count, Sleep) Minutes** | **% Increase from Imputation of wear Time** |
| --- | --- | --- | --- | --- | --- | --- | --- | --- |
| **Compliant wear days (with and without sleep)**  60-minute imputation intervals  No imputation | 3,729,900  3,522,935 | +5.9%  Referent | 2,816,340  6,543,15 | +330.4%  Referent | 1,254,780  1,073,579 | +16.9%  Referent | 3,816,000  3,599,992 | +6.0%  Referent |
| **Partial wear days**  60-minute imputation intervals  No imputation | 792,600  659,984 | +20.1%  Referent | 608,580  174,465 | +248.8%  Referent | 102,720  82,407 | +24.7%  Referent | 841,020    692,455 | +21.5%  Referent |

Compliant wear days: includes days with sleep and without sleep
